# The Gut Microbiota as a Therapeutic Target in IBD and Metabolic Disease: A Role for the Bile Acid Receptors FXR and TGR5

**DOI:** 10.3390/microorganisms3040641

**Published:** 2015-10-10

**Authors:** Annemarie Baars, Annemarie Oosting, Jan Knol, Johan Garssen, Jeroen van Bergenhenegouwen

**Affiliations:** 1 Nutricia Research, 3584 CT, Utrecht, The Netherlands; E-Mails: Annemarie.baars@danone.com (A.B.); Annemarie.oosting@danone.com (A.O.); jan.knol@danone.com (J.K.); johan.garssen@danone.com (J.G.); 2 Laboratory of Microbiology, Wageningen University, 6703 HB, Wageningen, The Netherlands; 3 Division of Pharmacology, Utrecht Institute for Pharmaceutical Sciences, Faculty of Science, Utrecht University, 3584 CG, Utrecht, The Netherlands

**Keywords:** gut microbiota, dysbiosis, FXR, TGR5, bile acid dysregulation, inflammatory bowel disease, metabolic disease, probiotics

## Abstract

The gut microbiota plays a crucial role in regulating many physiological systems of the host, including the metabolic and immune system. Disturbances in microbiota composition are increasingly correlated with disease; however, the underlying mechanisms are not well understood. Recent evidence suggests that changes in microbiota composition directly affect the metabolism of bile salts. Next to their role in digestion of dietary fats, bile salts function as signaling molecules for bile salt receptors such as Farnesoid X receptor (FXR) and G protein-coupled bile acid receptor (TGR5). Complementary to their role in metabolism, FXR and TGR5 are shown to play a role in intestinal homeostasis and immune regulation. This review presents an overview of evidence showing that changes in bile salt pool and composition due to changes in gut microbial composition contribute to the pathogenesis of inflammatory bowel disease and metabolic disease, possibly through altered activation of TGR5 and FXR. We further discuss how dietary interventions, such as pro- and synbiotics, may be used to treat metabolic disease and inflammatory bowel disease (IBD) through normalization of bile acid dysregulation directly or indirectly through normalization of the intestinal microbiota.

## 1. The Microbiome and Human Health

The human microbiota is established soon after birth and starts out as a dynamic ecosystem, subjected to large compositional shifts, that stabilizes and converges to a more “adult”-type of microbiota at 2–3 years of life [1,2]. The adult microbiota is highly complex with several hundred species-level phylotypes dominated by the phyla of *Actinobacteria*, *Bacteriodetes* and *Firmicutes* [3]. Analysis of microbiota samples from individuals from all over the world indicates a vast variation in bacterial taxa and high inter-individual variability in microbiota composition [4]. Despite the fact of this vast variation, metagenomic studies indicate a high functional redundancy between different microbiomes and suggest a shared core of functionalities between individuals [5]. Thus, characterization of intestinal microbial communities and metagenomic analysis are necessary to define a healthy human gut microbiome and to better understand the relationship between microbiota composition and disease susceptibility or disease progression. Therefore, defining a healthy human gut microbiome requires studies that not only address “who is there” but also “what are they doing” to link microbial composition with function in health and disease [6,7].

Infants receive their first bacterial inoculum via vertical transmission of components of the mother’s microbiome at birth, which is further reinforced by breastfeeding [8]. Internal and external factors that perturb this process such as mode of delivery (C-section *versus* vaginal), type of feeding (formula *versus* breast), antibiotic use (maternal or infant) or gestational age are suggested to impact microbial composition and therefore play a role in susceptibility to disease throughout life [1,8,9]. An abnormal shift in the microbiome compared to healthy individuals relates to changes in microbial communities and/or alterations in metabolic activity and is often referred to as dysbiosis. Dysbiosis is becoming increasingly appreciated as an environmental factor affected by host genetics, antibiotic use and diet. Moreover, dysbiosis is likely the cause or consequence of diseases located in the intestine such as ulcerative colitis [10] and inflammatory bowel disease [11] but also in systemic diseases such as chronic liver disease [12], atopic dermatitis [13], type 1 and 2 diabetes [14,15] and obesity [16].

Recent studies have identified a critical role for the intestinal microbiota in regulating digestion but also in regulating the development and function of innate and adaptive immunity [17,18,19,20]. However, much less is known how bacterial metabolites, such as deconjugated bile acids (BA) and secondary BAs, regulate host immunity. Accumulating evidence suggests a link between dysbiosis and pathological changes in the metabolism of BAs in patients suffering from obesity and type 2 diabetes [21], cardiovascular disease [22] and inflammatory bowel disease [23].

BAs play an important role in the digestion and absorption of dietary fats and fat-soluble vitamins and act as signaling molecules that regulate metabolic homeostasis through activation of BA receptors such as Farnesoid X receptor (FXR) and G protein-coupled BA receptor (TGR5). Besides being regulators of metabolic homeostasis, FXR and TGR5 are also expressed by cells belonging to both the innate and adaptive immune system suggesting a role for BAs in immune cell homeostasis and function. In this review, we discuss how the gut microbiota regulates BA homeostasis and how this impacts metabolic function and intestinal immune homeostasis through activation of FXR and TGR5. In the context of inflammatory bowel disease and metabolic disease, we discuss how intestinal dysbiosis and BA dysregulation contribute to disease pathogenesis. Lastly, we discuss how dietary interventions, such as pro- and synbiotics, could be used to restore BA dysregulation and alleviate IBD and metabolic disease.

## 2. Role of the Microbiome in BA Metabolism

In the gut, BAs are detergents required in the formation of mixed micelles, solubilization, digestion and absorption of lipids and fat-soluble vitamins from the intestine [24,25]. In the liver cholesterol is converted to primary BAs and conjugated to either taurine or glycine [26,27]. In the gallbladder, BAs are concentrated to bile composed of bile salts, water, phospholipids, cholesterol and electrolytes, minerals and bilirubin [25]. The total amount of bile that is circulating in the body is called the bile acid pool and consists for the larger part of the primary BAs cholic acid (CA) and chenodeoxycholic acid (CDCA) and their respective secondary BAs deoxycholic aicd (DCA) and lithocholic acid (LCA). Different from humans, in mice the major part of CDCA is converted to muricholic acid (MCA). Each BA has distinct biological activities and chemical properties. In response to meal ingestion, epithelial cells from the gut are triggered to secrete the hormone cholecystokinin leading to the release of conjugated BAs from the gallbladder [25]. BAs aid lipid absorption in the duodenum and are reabsorbed primarily in the distal ileum from where they travel back to the liver where they are recycled. The process of BA release, absorption and recycling in the liver is referred to as the enterohepatic circulation [28].

**Figure 1:**
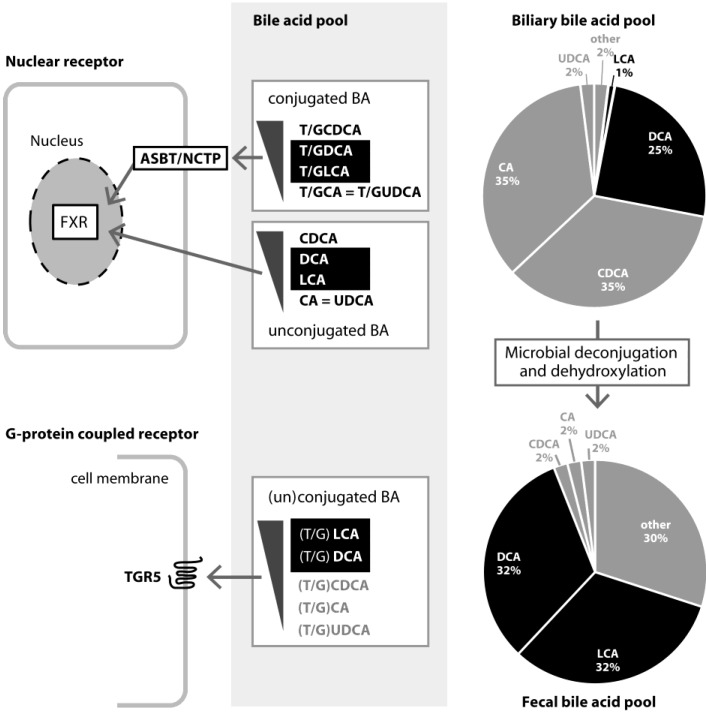
Composition of bile acids in the gallbladder and feces of healthy individuals and their signaling pathways.

Intestinal absorption of the BA pool is about 95% efficient which indicates that approximately 5% (0.3–0.6 g of BAs per day) of the BA pool eludes absorption and may undergo extensive modifications by the intestinal microbiota (Figure 1). BAs may undergo several types of biotransformations [29] but here we focus on deconjugation (removal of the amino acid component) and dehydroxylation (replacement of the hydroxyl group with a hydrogen). Deconjugation refers to the bile salt hydrolase (BSH) mediated enzymatic hydrolysis of the *N*-acyl amide bound linking bile acids to their amino acid conjugates and is thought to decrease the bactericidal activity of BAs. This reaction is substrate-limiting and takes place across the entire length of the intestinal tract and goes to completion in the colon.

A recent study by Sayin *et al*, has shown that, indeed, absence of an intestinal microbiota has profound effects on both the level and composition of the BA pool. Compared to conventional raised mice (CONV), germ-free mice (GF) have reduced levels of BAs in the cecum, colon and feces compensated by increased BA levels in the gallbladder and small intestine, leading to an overall increase in the BA pool. In addition, GF mice show a reduced diversity in BA species especially obvious due to the lack of unconjugated and secondary BAs [30].

The importance of bacterial BSH-activity in BA metabolism and directing local and systemic gene expression profiles in metabolic pathways was recently elegantly demonstrated by Jocye *et al* [31]. In this study, a BSH-negative *Escherichia coli* strain (EC) was modified to express a BSH gene derived from probiotic bacteria (ECBSH) and used to colonize germ-free mice. Plasma, liver and fecal concentrations of conjugated BAs were decreased following colonization with ECBSH compared to colonization with EC. Furthermore, expression profiles of genes involved in lipid digestion and absorption, adipocyte signaling, circadian rhythm and immune homeostasis were different in ileum and liver of mice receiving ECBSH compared to EC, which suggests that transcription of several pathways is affected by BSH expression. Finally, BSH improved metabolic phenotype because ECBSH supplemented mice showed reduced weight gain and lower plasma cholesterol compared to EC supplemented mice [31].

Genes coding for BSH have been identified and enzymes were isolated from several species of intestinal bacteria showing differences in gene regulation and organization, protein subunit size and composition, pH optimum, kinetic properties and substrate specificity [32,33]. Moreover, a very small specific subset of bacteria can further biotransform deconjugated BAs by 7αβ-dehydroxylation to produce secondary BAs like DCA and LCA [32]. Deconjugated BAs and secondary BAs are more lipophilic and membrane permeable and therefore more readily absorbed by epithelial cells lining the intestinal tract where they serve as signaling molecules for receptors such as FXR and TGR5 [25].

## 3. Role and Functions of FXR

Nuclear receptors (NR) are a large family of regulatory factors which, upon activation by hormonal, metabolic or nutrional signals, act as transcription factors controlling fundamental processes important for energy and metabolic homeostasis [34]. The NR FXR was originally described as the receptor for farnesol, an intermediate in the cholesterol synthesis pathway naturally derived from both plant and animal material [35]. Later on, it became clear that FXR is more appropriately characterized as a bile acid (BA) receptor with endogenous physiological concentrations of bile acids as its ligands (Figure 1) [36].

### 3.1. FXR and Bile Acid Metabolism

FXR is highly expressed in both liver and intestinal tissues [37]. In the intestine, FXR controls the absorption of BAs by regulating of the expression of proteins involved in uptake and transport of BAs from the intestine to the circulation. These include the apical sodium dependent transporter (ASBT) and the intracellular intestinal bile acid-binding protein (IBABP) that enable uptake and intracellular transport, respectively and the basolateral organic solute transporters α (OSTα) and β (OSTβ) which mediate BA efflux [38,39,40]. In the liver, FXR regulates OSTα en OSTβ, which, together with the Na-taurocholate cotransporting polypeptide (NTCP), stimulates hepatocytic uptake of BAs.

Signaling actions are mediated by nuclear receptor FXR and cell surface G-protein coupled receptor TGR5. Unconjugated and conjugated BAs that activate FXR are sorted by decreasing potency (top:high, bottom:low). Conjugated BAs require the presence of cell membrane expressed ABST (intestinal epithelial cells) or NTCP (liver cells) to initiate FXR activation. Conjugated as well as unconjugated BAs activate TGR5 and are sorted by decreasing potency (top:high, bottom:low). T = Taurine, G = Glycine; Primary BAs: CA = Cholic acid, CDCA = Chenodeoxycholic acid, UDCA = Ursodeoxycholic acid; Secondary BAs: DCA = Deoxycholic acid, LCA = Lithocholic acid.

Activation of the bile salt export pump (Bsep) by FXR drives export of BAs from the hepatocytes [37,38,39]. Moreover, liver FXR regulates the expression of the enzyme cholesterol 7 α-hydroxylase (CYP7A1) critically important as the rate-limiting enzyme in the synthesis of BAs from cholesterol [40]. Uptake of BAs in the intestine leads to activation of intestinal FXR and the expression and release of the protein fibroblast growth factor 19 (FGF19; FGF15 in mice). As a result, FGF19/15 circulates to the liver were it binds the hepatic FGF receptor 4 (FGFR4) in complex with β-Klotho. FGFR4-β-Klotho activation leads to suppression of the gene encoding CYP7A1 resulting in a suppressed BA synthesis. Overall, it can be concluded that FXR activation in the liver induces genes that impact bile acid pool size and composition while in the intestine FXR regulates genes that are involved in bile acid homeostasis [41].

How FXR expression is exactly regulated is still unclear, however, intestinal FXR expression seems to be regulated by the presence of microbiota. Compared to GF mice, CONV mice have an increased expression of intestinal FXR and its downstream targets, while liver FXR expression was similar [30]. Presence of microbiota seems to be sufficient for FXR gene induction as differences in intestinal microbiota composition does not seem to have an impact of FXR expression [42]. A recent study indicated that a 21-day treatment of CONV mice with the probiotic mixture VSL#3 was sufficient to induce significant changes in microbiota composition and bile acid homeostasis without changes in FXR expression [42]. Since bacteria are predominantly recognized by the family of Toll like receptors (TLRs), the previous argues for a role of TLRs in FXR expression. Indeed, activation of TLRs by synthetic agonist regulates expression of FXR, most likely via the induction of interferon regulatory factor 7 (IRF7) [43]. Next to regulation by IRF7, FXR expression in the intestine is also regulated by the caudal related homeobox 2 (Cdx2) protein, a transcription factor with a primary role during embryonic development. Indeed, knockdown of Cdx2 in epithelial cell lines abolishes FXR expression corroborated by the finding that in heterozygous Cdx2^+/−^ mice a reduced expression of intestinal FXR could be observed compared to wild-type mice [44]. Interestingly, depending on the dose and type of BAs, Cdx2 gene expression is either induced or repressed in epithelial cell lines [45]. This would suggest a complex feedback mechanism where BAs themselves modulate BA homeostasis via indirect regulation of the FXR receptor expression, however it remains to be determined whether modulation of CDX2 expression had a direct effect on FXR expression, as this was not researched [45]. Nevertheless, BAs regulate CDX2 expression, a process dependent on the transcription factor NFκB [45]. Since NFκB is highly involved in inflammatory processes, this would suggest that intestinal FXR expression might be susceptible to inflammatory conditions [46]. The relation between FXR and inflammation will be discussed more in detail in Section 3.4.

### 3.2. Role of FXR in Metabolic Homeostasis

Next to the well-established role of FXR in BA metabolism, FXR also regulates cholesterol, lipid and glucose metabolism (reviewed in [47,48]). Activation of FXR by a synthetic agonist was shown to improve hepatic steatosis, insulin sensitivity and reduced weight gain [49,50,51]. Ma *et al.* [50] suggested that FXR activation in mice fed a high fed diet inhibits hepatic fat accumulation by suppressing mRNA expression of CD36 which regulates fatty acid uptake. Moreover, the expression of genes related to hepatic lipogenesis was found to be reduced which blocks the development of hepatic steatosis. The underlying mechanism of FXR activation in insulin sensitivity is not known. Lipotoxicity can affect insulin signaling and by reducing plasma lipid levels via FXR function, insulin sensitivity can be ameliorated [52]. Previous findings also suggest that FXR function affects adipose tissue functionality since FXR activation improved weight loss. FXR was induced during adipocyte differentiation in 3T3-L1 and mouse embryonic fibroblasts (MEF) cells. Moreover, MEF cells from FXR knock-out (FXR-KO) mice revealed an impaired adipocyte differentiation [51]. These outcomes indicate that FXR is involved adipocyte function and thereby controlling metabolic homeostasis. Contradictory effects of FXR activation are also well-described. Watanabe *et al* [53] demonstrated that mice fed the synthetic FXR agonist GW4064 displayed enhanced weight gain and insulin resistance. In these mice lipid accumulation was increased in adipose tissue and liver, affecting both metabolic health and glucose intolerance. Consistent with these findings, FXR-KO mice were protected against diet induced obesity and showed improved glucose tolerance [54,55]. These contradictory effects of FXR activation on weight gain might potentially be explained by the use of synthetic *versus* natural agonists. Mice administered the synthetic FXR agonist GW4064 show a reduction in BA pool size, while administration with the natural FXR agonist CA led to an increased BA pool size [53,56]. These are relevant differences because an increase in BA pool size is associated with weight loss [57].

### 3.3. Role of FXR in Mucosal Protection

In addition to their role in lipid absorption, BAs affect the microbiota and intestinal integrity. Previous experiments have shown that obstruction of bile flow, e.g. during bile duct ligation (BDL), leads to bacterial overgrowth and mucosal injury, which can lead to bacterial translocation across the mucosal barrier into the circulation, potentially leading to systemic infection [58,59,60]. Further evidence comes from studies were it was shown that oral administration of BAs inhibits bacterial overgrowth and prevents bacterial translocation in situation of obstructed bile flow [60,61]. Moreover, *in-vitro* experiments indicated that BAs inhibit bacterial growth [33], potentially due to BA induced protein unfolding and aggregation preventing bacterial survival and colonization [62]. In general, BA susceptibility or tolerance is dependent on membrane architecture and composition and therefore a strain-specific trait and cannot be generalized [33]. A second physiological function of BAs in the prevention of bacterial overgrowth and intestinal epithelium integrity has recently been shown to involve FXR. Studies by Inagaki *et al.* indicated that FXR knockout mice (FXR-KO) have a reduced epithelial barrier integrity compared to wild-type (WT) mice, accompanied by a higher incidence of bacterial translocation. They further observed that WT or FXR-KO mice from which the bile ducts were ligated show similar defects in barrier function and bacterial translocation. The importance of FXR signaling in these observations was demonstrated by the finding that, in contrast to BDL FXR-KO mice, BDL WT mice were sensitive to administration of the FXR ligand GW4064, which improved barrier integrity and reduced bacterial translocation [63]. It is not clear how the enteroprotective effects of FXR are regulated, however, treatment of WT mice with GW4064 upregulates several genes in the intestine known to have antimicrobial properties such as *iNOS*, *IL18* and *Ang1* [63]. This suggest that at least part of the enteroprotective effect of FXR is to promote antimicrobial defense and epithelial integrity in the intestine [64]. A third biological function of BAs in the intestine concerns the role of FXR in epithelial cell proliferation. Mice deficient in FXR show an increased proliferation of colon cells, leading to an increase in size of intestinal adenocarcinomas in susceptible mice [65]. In agreement, analysis of human healthy tissue and adenocarcinomas indicates a loss of FXR function associated with colon cancer, suggesting that physiological activation of FXR controls has tumor-protective effects [66].

### 3.4. Role of FXR in Inflammation

NRs are well known to negatively regulate the activity of other signal-dependent transcription factors such as the activator protein 1 (AP-1) and nuclear factor κB (NFκB), a process depending on the induction of the nuclear co-repressors NCoR and SMRT [67]. The initial step of this process requires the recruitment and activity of the small ubiquitin-like modifier SUMO. SUMOylation of the NR targets the NR to NCoR thereby preventing clearing of the NCoR complexes from the promoter and as a result, target genes are maintained in a repressed state [68]. Recently it was shown that also FXR requires SUMOylation to regulate the expression of FXR target genes, suggesting that FXR might play a role in inflammatory processes via modulation of the transcriptional activity of AP-1 and or NFκB [46,69]. The first evidence indicating a role for FXR in inflammatory conditions was a set of experiments showing negative crosstalk between FXR and NFκB signaling pathways. Activation of FXR in liver cells was shown to inhibit the NFκB-mediated hepatic inflammatory response while on the other hand, hepatic NFκB activation suppressed FXR-mediated gene expression [70]. In agreement with the reciprocal actions of FXR and NFκB in the liver, experiments using intestinal cells indicated that NFκB directly interacts with FXR leading to a repressed FXR activity and the subsequent reduced expression of FXR target genes. Moreover, using a NFκB reporter assay, treatment with FXR agonists reduced TNFα induced NFκB activation [46,71,72]. Next to liver cells and intestinal epithelial cells, cells of the innate immune system are also sensitive to FXR-mediated inhibition of inflammation. Treatment of LPS stimulated human peripheral blood mononuclear cells, monocytes, macrophage and dendritic cells with a FXR agonist significantly reduced pro-inflammatory cytokine release [46,71]. Moreover, *in-vivo* treatment of mice with an FXR agonist significantly reduced inflammatory cytokine release from *ex-vivo* LPS stimulated mononuclear cells obtained from mouse peritoneal exudate fluid [71]. This effect was also observed using isolated laminar propria mononuclear cells (LPMCs) from inflammatory bowel disease (IBD) patients. Presence of an FXR agonist during antibody cocktail-mediated restimulation of LPMCs led to a reduced release of IFNγ, IL-17 and TNFα compared to the absence of the FXR agonist [71]. Based on these results, it can be anticipated that absence of FXR would lead to a more severe intestinal inflammation. Indeed, compared to WT mice, FXR KO mice have an increased inflammatory phenotype characterized by an increased expression of pro-inflammatory and profibrogenetic genes in the colon, increased number of lamina propria CD11b^+^ cells and enhanced production of pro-inflammatory cytokines following LPS stimulation of lamina propria isolated CD11b^+^ cells [46]. As a result, FXR KO mice are more susceptible and show an increased inflammatory phenotype in trinitrobenzenesulfonic acid (TNBS) or dextran sulfate (DSS)-induced models of intestinal inflammation compared to WT mice [46]. Moreover, treatment of WT, but not FXR KO mice, with an FXR agonist could protect against intestinal inflammation, demonstrating that FXR activation protects against the development of intestinal inflammation in murine models of inflammatory bowel disease [46,71]. In the human setting, further support for a role for FXR in intestinal inflammation comes from examination of FXR expression in tissues from Crohn’s disease patients. Both in the ileum and colon of patients a decreased expression of FXR and its target genes could be observed [46,73].

## 4. Role and Functions of TGR5

TGR5 belongs to the family of G-protein coupled receptors (GPCRs). GPCRs are a large family of cell surface receptors expressed in many different organs and cells. They recognize a vast array of ligands and play a role in a wide variety of physiological processes such as visual and taste sense, behavioral and mood regulation, activity of the immune system and inflammation and regulation of sympathetic and parasympathetic nerve signaling. There are currently three known GPCRs that are show to be activated by BAs; TGR5 (also known as M-BAR or GP-BAR1), muscarinic receptors [74] and formyl-peptide receptors [75]. The best-studied GPCR in relation to BA metabolism is TGR5. TGR5 is highly expressed in intestinal tissues and immune cells and is activated by both conjugated as well as unconjugated BAs with highest affinity for secondary BAs (Figure 1) [76,77].

### 4.1. TGR5 and BA Metabolism

The exact role of TGR5 in BA metabolism remains to be elucidated. However, TGR5 knockout (TGR5 KO) mice have reduced levels of circulating BA compared to WT mice suggesting a role for TGR5 in BA homeostasis [78,79]. The mechanism underlying this difference in BA pool is unknown, but increased excretion of BA in the feces was not apparent in TGR5-KO mice and thus an unlikely cause [78]. One of the mechanisms that could lead to a reduction in BA pool size is increased activation of the negative feedback regulation of BA synthesis via FGF19/15. TGR5 KO mice show a decreased amount of tauro-β-muricholic acid (βMCA) with similar CA levels [79]. βMCA functions as an antagonist of FXR and an increased ratio of CA to βMCA leads to increased FXR activation and FGF19/15 release which would inhibit de-novo synthesis of BA from cholesterol and thus a reduced BA pool [30]. However, the direct link between TGR5 activation and the reduction of βMCA remains to be established.

### 4.2. Role of TGR5 in Metabolic Homeostasis

The TGR5 receptor has been known to be a specific receptor for bile acids (reviewed in [80]). TGR5 activation by BAs stimulates the release of GLP-1 in intestinal cells, affecting indirectly the insulin secretion in pancreatic β- cells and thereby impacting insulin sensitivity [81]. Moreover, BAs can also directly stimulate glucose-mediated insulin secretion, because pancreatic β-cells also express TGR5 receptors [82]. These findings demonstrate a link between BAs and TGR5 in controlling glucose metabolism. However, it is important to take gender differences into account when studying the link between TRG5 and metabolic health as male TGR5 KO mice challenged with a high fed diet showed diminished insulin sensitivity, while female mice displayed an improved insulin sensitivity [80].

TGR5 also plays a role in atherosclerosis as TGR5 activation in CONV mice was found to reduce macrophage inflammation and lipoprotein uptake resulting in less atherosclerotic plaque formation, which decreases the development of atherosclerosis [83]. Moreover, TGR5-mediated nitric oxide (NO) production by vascular epithelial cells suppresses monocyte adhesion, which is a prerequisite for the development of atherosclerosis [84].

### 4.3. Role of TGR5 in Mucosal Protection

Complementary to the role of FXR in the maintenance of barrier integrity, TGR5 has also been shown to play a role in intestinal homeostasis. TGR5 KO mice were shown to have an abnormal morphology of the colonic mucous and an increased intestinal permeability compared to WT mice, which might underlie their increased susceptibility to colitis [85]. An important observation in this study is that despite TGR5 expression by intestinal epithelial cells, a significant increase of TGR5 expression in the inflamed colon occurs primarily in the infiltrating mononuclear cells [85]. Moreover, no differences in colonic histopathology could be observed between TGR5 WT and TGR5 KO animals until 12 months after birth, suggesting that the protective effect of TGR5 might be primarily mediated via effects on the innate cell population [85]. In agreement, lamina propria isolated mononuclear cells (LPMCs) isolated from inflamed tissue of Crohn’s disease patients have a significant increased expression of TGR5 compared to either LPMCs from non-inflamed tissue or healthy controls and are more prone to release inflammatory mediators upon stimulation. Importantly, treatment of LPMCs with TGR5 agonists suppressed inflammatory cytokine production after bacterial stimulation [86]. Overall, this suggests that TGR5 contributes to maintain intestinal homeostasis primarily via prevention or attenuation of inflammatory responses.

### 4.4. Role of TGR5 in inflammation

One of the initial studies on TGR5 indicated the presence of TGR5 on immune cells, mainly on monocytes and macrophages. Upon triggering of TGR5 with BAs, an increase in the intracellular levels of the second messenger cyclic AMP (cAMP) was measured [76]. Since it was previously established that cAMP expression could suppress LPS-stimulated cytokine production by macrophages, these data suggest that BAs can suppress macrophage pro-inflammatory cytokine release at least in part due to activation of TGR5 and the subsequent increase in cAMP [87]. Indeed, BA stimulation of TGR5 transfected monocytes could inhibit LPS-induced pro-inflammatory cytokine release while non-transfected monocytes remained refractory to BA stimulation [76]. Moreover, BA mediated TGR5 activation of macrophages was shown to attenuate activation of NFκB, an effect that could be blocked using an inhibitor of protein kinase A (PKA). This suggests that TGR5-cAMP-PKA activation inhibits NFκB activity, most likely via kinase-anchoring proteins [88,89]. In contrast to the anti-inflammatory actions of TGR5 in inflammatory conditions, activation of TGR5 in the absence of inflammation induces inflammatory cytokines such as IL-1β and TNFα in innate cells [90]. Although this dual action of TGR5 is not entirely understood, it might serve to protect against potential toxic effects of BA when they reach abnormally high levels. BA-activated liver Kupffer cells release increased levels of pro-inflammatory cytokines, which suppress Cyp7a1 expression and in doing so initiate a negative feedback mechanism most likely mediated via TGR5 [88,90]. This hypothesis is strengthened by the finding that TGR5 activation is crucial against BA overload after partial hepatectomy [91].

Further evidence for a regulatory role of TGR5 in inflammation comes from *in vivo* studies. *In vivo*, mice deficient in TGR5 produce a significant higher level of inflammatory serum markers compared to WT mice after a systemic challenge with LPS. Moreover, livers from TGR5 KO mice show increased levels of infiltrating inflammatory cells, primarily due to the influx of F4/80^+^ macrophages [92]. Treatment of WT mice, but not TGR5 KO mice, with a TGR5 BA agonist attenuated the inflammatory response, linking BA-mediated TGR5 activation to systemic anti-inflammatory effects [92]. In an elegant set of experiments, Perino *et al.* further confirmed an *in vivo* immunoregulatory role for TGR5 expressed on macrophages [93]. Using chimeric mice, carrying either TGR5^−^ or TGR5^+^ leukocytes, it was shown that high fat diet induced adipose tissue inflammation was exacerbated in the TGR5^−^ mice. A prominent role for TGR5 on macrophages was confirmed by using mice specifically deleted for TGR5 in the macrophage population (LysM-Cre *Tgr5*^fl/fl^ mice). Both chimeric TGR5^–^ and LysM-Cre *Tgr5*^fl/fl^ mice were characterized as having an elevated adipose tissue inflammatory phenotype due to the increased influx of M1 macrophages and enhanced expression of chemokines [93]. Since macrophage inflammation is central to many types of chronic inflammatory diseases, increasing the levels of TGR5 agonistic BAs holds promise in health and disease [80].

## 5. FXR and TGR5 as Therapeutic Targets

**Figure 2:**
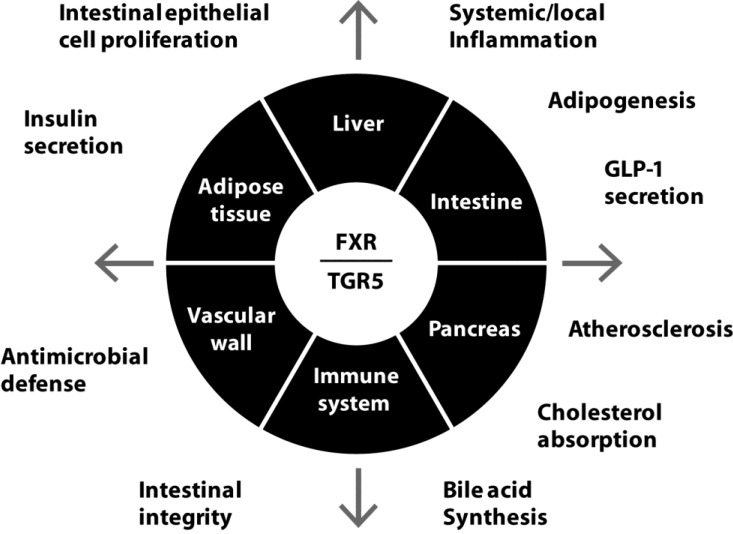
Schematic overview of the expression and functions of FXR and TGR5. See Section 3 and Section 4 for details. White on black; Tissue or cellular expression of FXR and TGR5. Black on white; Functions of FXR and TGR5.

Due to their widespread expression in many different types of cells and tissue and their important role in many physiological processes such as bile acid, lipid, carbohydrate and cholesterol metabolism as well as inflammation and intestinal homeostasis, FXR and TGR5 are both increasingly recognized as targets for drug development (Figure 2) [94,95]. Over the years, several steroidal (*i.e*., BA analogues) as well as non-steroidal agonists have been development and tested in preclinical models of disease. The most widely investigated steroidal FXR and TGR5 agonists are; 6α-ethyl-chenodeoxycholic acid (INT-747 or obeticholic acid (OCA)), 6alpha-ethyl-23(S)-methylcholic acid (INT-777) and 6α-ethyl-3α,7α,23-trihydroxy-24-nor-5β-cholan-23-sulfate (INT-767). GW4064 is the best-known non-steroidal agonist. It is beyond the scope of this review to provide detailed information on the effects of these agonists on human health but for a recent overview we refer to a review by Schaap *et al.* [95].

Instead of the pharmaceutical treatment with specific BAs or synthetic BA agonists, an alternative therapeutic strategy could entail interventions at the microbial level. Since the intestinal microbiome regulates the BA homeostasis, changes in the microbiota due to diet or disease may impact both BA pool size and composition and therefore physiological processes mediated by FXR and TGR5. In the next sections we discuss how changes in the composition of the microbiota in IBD and metabolic disease contribute to BA dysregulation and how alterations in FXR and TGR5 activation might play a role in disease pathogenesis. Subsequently, we propose dietary interventions with pro or synbiotics as a natural therapeutic method to normalize both microbiota composition as well as BA homeostasis.

## 6. Dysbiosis, BA Dysregulation and Inflammatory Bowel Disease

IBD patients, which include patients suffering from ulcerative colitis and Crohn’s disease, are diagnosed with a remitting and relapsing condition characterized by patches of chronically inflamed tissues at various sites throughout the gastrointestinal (GI) tract. Microbiota analysis of IBD patients indicate an overall decrease in biodiversity with a specific decrease in bacteria from the *Bacteroidetes* and *Firmicutes* phylum, increased amounts of Gammaproteobacteria species and the prevalence or absence of specific bacteria [96,97,98,99]. Among other potential mechanisms, these changes in microbiome populations may lead to alterations in BA metabolism. The majority of BSH activity in the GI tract comes from bacteria belonging to the phylum *Firmicutes* (30%), *Bacteroidetes* (14.4%) and *Actinobacteria* (8.9%) [100]. Thus, a decrease in *Bacteriodes* and *Firmicutes* might underlie the alterations in BA metabolism observed in IBD patients. Metagenomic analysis of bacterial bile metabolizing-gene abundance in IBD patients indicates a loss in the abundance of BSH genes [101]. Moreover, a reduced enzymatic activity was observed in IBD patients resulting in impaired deconjugation, dehydroxylation and desulfation of BAs [23]. Consequently, a reduction in total secondary BA could be observed in both serum and feces while the conjugated BAs in feces were increased [23]. Loss of unconjugated and secondary BAs could lead to a reduced activation of FXR and TGR5 and therefore a loss of anti-inflammatory actions and gut integrity protecting effects, which contributes to the pathogenesis of IBD [23]. In support of this hypothesis, genetic variation in the gene coding for FXR (*NR1H4*) is found to be associated with IBD. Five single nucleotide polymorphisms (SNP) in the *NR1H4* gene were investigated of which the SNP *rs3863377* showed an inverse association with IBD [102]. Although it is currently unclear whether this SNP leads to a loss or gain of function of FXR, the IBD population has significantly lower frequency of carriers for SNP *rs386377* compared to the healthy population, suggesting a protective effect of SNP *rs386377* against the disease [102]. Further support comes from a study that showed that expression of FXR and its target genes are decreased in the intestine of Crohn’s disease patients [73].

## 7. Dysbiosis, BA Dysregulation and Metabolic Disease

Metabolic syndrome (MS) can be described as a combination of several metabolic and cardiovascular risk determinants which together increase the risk of metabolic diseases such as diabetes and cardiovascular disease [103]. Central to MS pathology are obesity, a chronic systemic low-grade inflammatory state and ectopic fat accumulation, which is currently thought to be due to disturbed regulation of key metabolic genes. Accumulating evidence suggest that environmental factors such as maternal nutrition, body composition and stress hormone levels might underlie MS predisposition starting in early life ranging from the developmental period until the introduction of solid foods [104,105,106,107]. The gut microbiota is increasingly being accepted as a factor that effects host metabolism with *Bacteroidetes* and *Firmicutes* playing important roles in nutrient absorption, mucosal barrier integrity and postnatal intestinal maturation [108,109,110,111]. The nature of dysbiosis associated with metabolic disease is currently unclear. In contrast to obvious differences in the case of inflammatory bowel disease, dysbiosis in obesity does not display a consistent signature [112]. For instance, a wealth of data support an increased ratio of *Firmicutes* to *Bacteroidetes* to be associated with obesity [16,113] but other studies show no trend or even the opposite [114,115,116]. On a microbiome level, comparison of gene number across obese and non-obese subjects show that low microbiome gene richness is associated with MS determinants such as adiposity, insulin resistance and dyslipidemia [117,118]. The gut microbiome, which includes the microbiota, their environmental interactions and their genetic information, is 150 times larger as contained within the human genome [119]. This indicates that for dysbiosis associated with metabolic disease research should not only focus on microbiota composition but also on microbiota expressed genes and metabolic activity. Differences in body composition and microbiome appear to correlate to differences in BA metabolism. Currently, bariatric surgical procedures including vertical sleeve gastrectomy (VSG) or Roux-en-Y gastric bypass (RYGB) are most effective in the treatment of obesity [120,121]. For example, VSG procedure resulted in improved glucose tolerance and weight loss [122]. These metabolic improvements are linked to changes in FXR signaling, circulating bile acids and alterations in the gut microflora [122]. Bariatric surgical procedures are associated with increased circulating bile acids impacting the enterohepatic bile acid circulation. Previous findings demonstrated that transplantation of the microbiome from obese mice into germ free mice was linked to body weight increase [123]. However, transplantation of the gut microbiome from bariatric surgical mice into germ free was linked with body weight reduction [123]. Clinical outcomes showed similar effects as bariatric surgical treatment in obese people resulted in a more lean like microbiota profile [120].

Fecal transfers of feces from female adult twins discordant for obesity into germ-free mice resulted in significantly lower levels of eight out of 37 BA species, suggesting a reduced capacity of BA transformation in mice receiving the “obese” microbiota [124]. Moreover, due to an increased release of FGF15 in the circulation combined with a reduced expression of liver Cyp7a1 expression in obese *versus* lean mice, a reduced synthesis of BA was reported [124]. As previously mentioned, *Firmicutes*, which are gram-positive bacteria, are the predominant bacteria able to deconjugate and dehydroxylate primary BAs into secondary BAs. In a recent study, male obese subjects who received vancomycin (antibiotic which acts on gram-positive bacteria) but not amoxicillin (antibiotic which acts on gram-negative bacteria) showed a decreased microbiota diversity [125]. Further analysis of BA composition indicated that only treatment with vancomycin reduced levels of secondary BAs with a concomitant increase in fecal primary BAs and this change in BA metabolism was correlated with decreased peripheral insulin sensitivity [125].

BA dysregulation results in increased intestinal permeability and intestinal inflammation, factors that contribute to systemic low-grade inflammation which is associated with insulin resistance and obesity [26]. Poorly regulated levels of cholesterol and triglycerides as well as inflammation of the arterial walls increase the risk for cardiovascular disease (CVD). Among other factors, recent data suggest a significant role for the gut microbiome in the pathogenesis of CVD [126,127]. Experiments done with germ-free mice have shown that absence of a microbiota leads to reduced intestinal cholesterol absorption and increased fecal cholesterol excretion [128]. In agreement, germ-free mice display reduced levels of plasma cholesterol [30]. While these data indicate the importance of the microbiota in cholesterol uptake, recent data also suggest that intestinal cholesterol also impacts microbiota composition. Compared to conventional mice, mice deficient in the intestinal cholesterol transporter Niemann–Pick C1-Like 1 (NPC1L1) display increased *Firmicutes* and decreased *Bacteroidetes* [128]. A similar decrease in *Bacteroidetes* and increased *Firmicutes* is also associated with obesity [111]. The major route of elimination of cholesterol is through the oxidative conversion to bile acids in the liver, however, presence of intestinal microbiota has a greatly accelerating effect presumably due to BSH-mediated BA elimination [129,130]. Based on this premises BSH-active bacteria have been used in clinical studies as treatment for CVD. In these studies BSH-active bacteria were shown to be efficient in lowering total and low-density lipoprotein cholesterol however, further studies are required to determine the exact mechanism of action [131].

## 8. Dietary Interventions with Pro or Synbiotics

One of the potential mechanisms of action of probiotics or the combination of probiotics with prebiotics (synbiotics) is their capacity to correct dysbiosis. A recent systematic review covering a total of 63 randomized clinical trials indicated that specific strains of probiotics, or specific combination of bacterial strains with or without prebiotics, were able to impact on the microbiota. Supplementation with probiotics was found to restore the microbiota after disruption, or to alter the microbiota in a state of dysbiosis, or to induce compositional shifts in a non-disrupted microbiota. Moreover, those probiotics that were able to impact the restoration of the normal microbiota were associated with the strongest strength of clinical efficacy [132]. Recent evidence suggests that probiotics, through modification of the microbiota, also impact BA metabolism. Supplementing healthy mice with probiotics altered the ratio of *Firmicutes* to *Bacteroidetes* and increased fecal BSH-expression and enzyme activity. This increase in BSH activity led to a decreased ratio of conjugated *versus* deconjugated BA species ultimately leading to increased fecal BA excretion and increased BA synthesis [42].

Probiotic bacteria are most often derived from the genera *Lactobacillus* and *Bifidobacterium*. In a large study screening over 300 strains of *Bifidobacterium* and *Lactobacilli* and the species *Lactococcus lactis* (*L. lactis*), *Leuconostoc mesenteroides* (*L. mesenteroides*) and *Streptococcus thermophiles* (*S. thermophiles*), BSH activity was found in 273 strains of *Bifidobacterium* and *Lactobacilli* while missing in *L. lactis*, *L. mesenteroides* and *S. thermophiles*. Moreover, nearly all bifidobacteria have BSH activity while this activity can be found only in selected species of lactobacilli [133]. It has been demonstrated that BSHs recognize BAs on both the amino acid groups (glycine/taurine) and the cholate steroid nucleus. Therefore, different probiotics were found to hydrolyze specific BA substrates. Moreover, because different BSHs might respond to different BA compositions many strains of probiotics possess more than one BSH homologue (reviewed in [134]).

A decrease in BSH-active bacteria is clearly linked to the dysbiosis as observed in IBD as well as IBS patients. As a result, both IBD and IBS patients show an abnormal BA metabolism [23,135]. One possibility to restore normal BA metabolism in these patients could be the introduction of highly BSH-active probiotics. Although supplementation with lactobacilli and bifidobacteria shows some merit in clinical studies involving IBD patients, data shows that the results are highly variable and strain-specific [132,136,137]. However, to our knowledge, none of the probiotics used in these studies were selected with the specific intention to address abnormal BA metabolism. Based on the beneficial effect of FXR and TGR5 agonists in pre-clinical studies on IBD [71,138,139], it would be interesting to investigate whether supplementation of probiotics with high-BSH activity correlates with clinical efficacy in previous studies or warrant their use in future clinical studies. In support, a recently conducted clinical study evaluating the effects of a BSH-active strain of *L. reuteri* in hypercholesterolemic adults, indicated that a significant higher proportion of the *L. reuteri* treatment group reported improved symptoms in IBS and unspecified bowel disorders with a reduction in markers for systemic inflammation [140,141].

Although lifestyle changes remain the primary therapy for metabolic syndrome and the related obesity, current therapies included strategies to lower plasma cholesterol and triglyceride levels. Accumulating evidence suggest that probiotics are effective in reducing plasma cholesterol and triglyceride levels by means of several different mechanisms (reviewed in [142]). (1) BSH-active probiotic strains deconjugate BAs. This reaction leads to decreased solubility of BAs, leading to a reduced reabsorption and increased excretion of BAs in feces. To maintain BA homeostasis, de-novo synthesis of BAs would lead to a reduction in plasma cholesterol levels. In addition, deconjugated BAs are more hydrophobic which could lead to a reduced cholesterol absorption in the intestine due to inhibition of cholesterol micelle formation [143]. (2) Probiotics bind cholesterol onto bacterial cell walls. Specific strains of probiotics were shown to assimilate cholesterol in their membranes thereby preventing cholesterol metabolic degradation. (3) Precipitation of cholesterol. Lowering of the pH due to scFA release, can lead to precipitation of cholesterol. (4) Conversion of cholesterol to coprostanol. Probiotic bacteria that produce cholesterol reductase convert cholesterol into the less soluble coprostanol leading to reduced intestinal absorption. (5) Production of short-chain fatty acids (scFAs). Propionate has been shown to reduce cholesterol synthesis, while acetate has the opposite effect. (6) Reduction of cellular cholesterol uptake. Direct interaction of probiotics with intestinal epithelial cells may lower expression of cholesterol transporters such as NPC1L1. Based on these potential mechanisms, a wealth of clinical studies have now shown efficacy of BSH-active probiotics in lowering cholesterol and triglyceride levels [131]. However, detailed investigation of probiotic BSH activity is needed as probiotics express different BSHs and different BSHs can have different effects on weight gain and plasma lipid levels [31]. It would be interesting to investigate whether these differences in probiotic BSH activities might contribute to the effects on weight loss or gain observed after probiotic treatment [144].

## 9. Conclusions and Perspective

In this review, we have discussed how the microbiota in the gastrointestinal tract regulates the metabolism of BA species. Microbial enzymes modify BAs and the resulting BA metabolites impact both metabolism as well as host immunity. The composition of the microbiota is causatively related to BA pool size and BA species diversity and perturbations of the microbiota leads to BA dysregulation as observed in IBD and metabolic disease.

With the discovery of receptors (e.g., FXR and TGR5) that recognize specific BAs, the field of research and application now incorporates both local and systemic inflammatory processes next to processes related to digestion and metabolism. BA receptors are not only expressed by tissues involved in BA metabolism such as the liver, gallbladder and intestine, but also by cells from the innate and adaptive immune system and the overall inhibitory effects on systemic inflammation suggests the importance of maintaining BA homeostasis [145].

**Figure 3:**
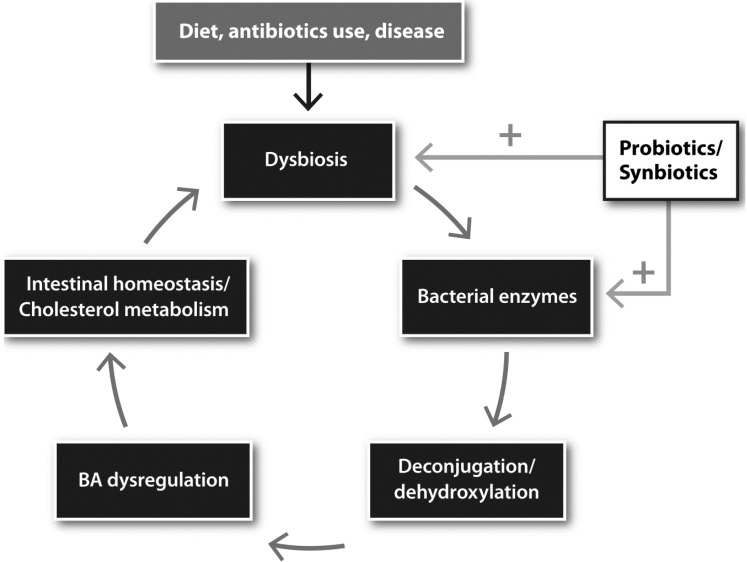
Schematic representation of the vicious cycle that follows the pathological perturbation of the intestinal microbiota.

Dysbiosis is often associated with a loss of BSH-expressing bacteria, which impacts host-microbe interactions and influences immune homeostasis, cholesterol metabolism and host weight gain. In this setting, BSH-expressing probiotics offer attractive natural means of normalizing BSH activity both directly, via supplementing lost BSH-activity, or indirectly through normalization of the intestinal microbiota (Figure 3).

Diet, antibiotic use and/or disease are factors that may cause disturbances in the gut microbiota composition. Changes in microbiota composition impact on BA pool size and composition through alterations in deconjugation and dehydroxylation activities. The subsequent change in BA metabolites directly and indirectly, through BA receptors FXR and TGR5, modulate dietary fat and vitamin metabolism, epithelial barrier integrity and intestinal immune homeostasis. Loss of barrier integrity leads to inflammatory processes that contribute to dysbiosis. Dietary interventions with probiotics or synbiotics can be used to change or alleviate the vicious circle. Probiotics may be used to treat dysbiosis by normalizing the gut microbiota composition or BSH-expressing probiotics may be used to supplement lost BSH-activity.

Future challenges include interrogation of the precise mechanisms through which pathological changes in microbiota composition leads to BA dysregulation. Finally, future research should investigate the molecular and biological activities of the different BSH-enzymes to achieve a more functional understanding of BSH activity. Elaborating our understanding of the microbiota composition-BA metabolism-immune system axis in health and disease may allow for the rational selection of BSH-expressing probiotics or synbiotics as preventive or therapeutic dietary interventions in inflammatory and metabolic disorders.
